# An Overview of Intracranial Ependymomas in Adults

**DOI:** 10.3390/cancers13236128

**Published:** 2021-12-05

**Authors:** Giuseppe Lombardi, Alessandro Della Puppa, Marco Pizzi, Giulia Cerretti, Camilla Bonaudo, Marina Paola Gardiman, Angelo Dipasquale, Fabiana Gregucci, Alice Esposito, Debora De Bartolo, Vittorina Zagonel, Matteo Simonelli, Alba Fiorentino, Francois Ducray

**Affiliations:** 1 Department of Oncology, Oncology 1, Veneto Institute of Oncology IOV-IRCCS, 35128 Padua, Italy; giulia.cerretti@iov.veneto.it (G.C.); vittorina.zagonel@iov.veneto.it (V.Z.); 2 Department of Neurosurgery, Department of Neuroscience, Psychology, Drug Area and Child Health (NEUROFARBA), University of Florence, Careggi University Hospital, 50134 Florence, Italy; alessandro.dellapuppa@unifi.it (A.D.P.); camilla.bonaudo@unifi.it (C.B.); alice.esposito@unifi.it (A.E.); 3 General Pathology and Cytopathology Unit, Department of Medicine-DMED, University of Padua, 35121 Padua, Italy; marco.pizzi.1@unipd.it (M.P.); marinapaola.gardiman@aopd.veneto.it (M.P.G.); deboradebartolo@unipd.it (D.D.B.); 4 Department of Biomedical Sciences, Humanitas University, 20090 Pieve Emanuele, Italy; angelo.dipasquale@cancercenter.humanitas.it (A.D.); matteo.simonelli@hunimed.eu (M.S.); 5 IRCCS Humanitas Research Hospital, 20089 Rozzano, Italy; 6 Radiation Oncology Department, Advance Radiation Therapy, General Regional Hospital F. Miulli, 70021 Acquaviva delle Fonti, Italy; fabianagregucci@gmail.com (F.G.); a.fiorentino@miulli.it (A.F.); 7 Lyon University, Université Claude Bernard Lyon 1, 69007 Lyon, France; francois.ducray@chu-lyon.fr; 8 Cancerology Research Center of Lyon, INSERM U1052, CNRS UMR 5286, Cancer Cell Plasticity Department, Transcriptome Diversity in Stem Cells Laboratory, 69007 Lyon, France; 9 Service of Neuro-Oncology, Hospices Civils de Lyon, Groupement Hospitalier Est, Neurology Hospital, 69007 Lyon, France

**Keywords:** ependymoma, brain tumors, glioma, chemotherapy, radiotherapy

## Abstract

**Simple Summary:**

Ependymomas are neuroepithelial tumors arising from the central nervous system. They can form anywhere along the neuraxis. In adults, these tumors predominantly occur in the spine. Local therapy with surgery and radiotherapy represents the most effective treatment while systemic chemotherapy should be used in recurrent cases. However, in recent years, a deeper knowledge of molecular mechanisms of these tumors has been made, allowing for new potential systemic treatments. Here, we review these treatment approaches and provide an overview on the molecular characteristics of ependymomas.

**Abstract:**

Ependymomas are rare primary central nervous system tumors. They can form anywhere along the neuraxis, but in adults, these tumors predominantly occur in the spine and less frequently intracranially. Ependymal tumors represent a heterogenous group of gliomas, and the WHO 2016 classification is based essentially on a grading system, with ependymomas classified as grade I, II (classic), or III (anaplastic). In adults, surgery is the primary initial treatment, while radiotherapy is employed as an adjuvant treatment in some cases of grade II and in all cases of anaplastic ependymoma; chemotherapy is reserved for recurrent cases. In recent years, important and interesting advances in the molecular characterization of ependymomas have been made, allowing for the identification of nine molecular subgroups of ependymal tumors and moving toward subgroup-specific patients with improved risk stratification for treatment-decisions and future prospective trials. New targeted agents or immunotherapies for ependymoma patients are being explored for recurrent disease. This review summarizes recent molecular advances in the diagnosis and treatment of intracranial ependymomas including surgery, radiation therapy and systemic therapies.

## 1. Introduction

Ependymomas are neuroepithelial tumors of the central nervous system (CNS). Typically, they are believed to arise from the ependymal lining of the ventricles, cerebral hemispheres and central canal of the spinal cord. Intracranial ependymomas are rare primary tumors, accounting for 2.5% of all intracranial gliomas and 7% of primary central nervous system tumors diagnosed annually [1,2]. They account for 5–12% of brain tumors in children and 1–3% of brain tumors in adults [1,3,4]. The incidence of ependymomas is estimated to be 0.43 patients per 100,000 population [1,2].

Ependymomas are basically classified by the World Health Organization (WHO) as grade I, II, or III (anaplastic). A distinction may also be made based on the site of origin: supratentorial or infratentorial tumors, given that anaplastic variants are more prevalent in the supratentorial region [5]. Grade I and II ependymomas are characterized by small size and slow growth, while anaplastic tumors develop at a much higher proliferative rate and often spread to other locations in the intracranial hemisphere through cerebrospinal fluid (CSF). Other uncommon intracranial locations have been described in the literature (extra-axial petroclival region, sellar region, pontocerebellar angle with involvement of the cavernous sinus, and pineal gland) [6,7,8,9,10]. Even though the majority of intracranial ependymomas begin from the ependymal cells of the cerebral ventricles and choroid plexus, they can also be found in the brain parenchyma, where they originate from heterotopic ependymal cell rests deposited during embryological development [11]. Patients with infratentorial ependymomas generally have a slightly more favourable prognosis than those with supratentorial ependymomas [12,13,14]. Younger age at diagnosis, a high tumor grade, and a large tumor size are associated with poor survival [15,16]. Spinal seeding occurs in approximately 10% of patients. In less than 5% of patients, seeding is present at the time of the initial diagnosis. Seeding is most likely to occur from tumors of the IV ventricle or from anaplastic ependymomas. Disseminated disease is actually more common in pediatric patients than in adults. 5-year and 10-year overall survival rates are around 83% and 79%, respectively [1].

## 2. Histology, Molecular Characteristics and Liquid Biopsy

### 2.1. Histology and Grading of Ependymal Tumors

Ependymal tumors are a heterogeneous group of gliomas, whose molecular features have been extensively defined in recent years. These studies have dramatically changed our understanding of these tumors, prompting major changes in their classification and prognostic stratification.

**Figure 1:**
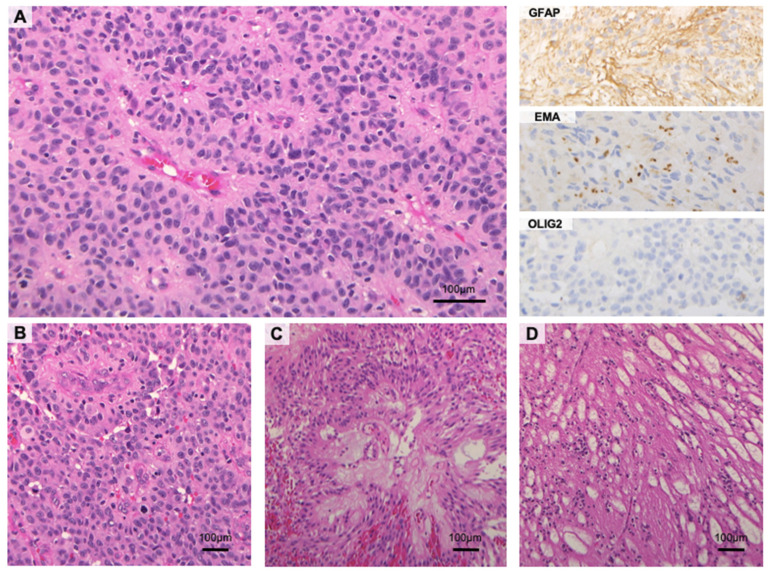
Histological features of ependymal tumors. (**A**) Classic ependymomas are well-circumscribed neoplasms with clear-cut vascular pseudorosettes. Immunohistochemically, the neoplastic cells show variable expression for GFAP with characteristic para-nuclear dot-like positivity for EMA. OLIG2 is weak to negative (**B**) Anaplastic ependymomas are hypercellular tumors with brisk mitotic activity, frequent micro-vascular proliferation, and palisading necrosis. (**C**) Myxopapillary ependymomas feature well-differentiated cuboidal to elongated tumor cells that are radially oriented around vascularized myxoid cores with a papillary architecture. Endothelial proliferation and cellular atypia are typically absent. (**D**) Subependymomas consist of small clusters of cells with isomorphic nuclei, scattered throughout a finely fibrillary background with microcysts (H and E and immunoperoxidase stains; original magnification, 10×, 20× and 40×).

The traditional classification of ependymal tumors is based on histological criteria and includes: (i) classic ependymoma (EPN), (ii) anaplastic EPN, (iii) myxopapillary EPN, and (iv) subependymoma (SE) [4]. Classic EPNs are well-circumscribed neoplasms, characterized by uniform small cells with perivascular pseudorosettes or (more rarely) ependymal rosettes. Pseudorosettes are perivascular anuclear zones formed by tumor cell processes, whereas ependymal rosettes consist of tumor cells surrounding a central rounded or elongated lumen, reminiscent of ependymal canals (Figure 1). Specific architectural and/or cytological features identify three EPN variants, referred to as papillary, clear cell (i.e., oligodendrocyte-like), and tanycytic (i.e., spindle cell-shaped) EPN. Classic EPNs may feature highly cellular areas, dystrophic calcifications, and/or foci of ischemic necrosis. A high nuclear-to-cytoplasmic ratio, brisk mitotic activity, palisading necrosis, and/or microvascular proliferation are not features of classic EPN and prompt the histological diagnosis of anaplastic EPN [4]. On immunohistochemistry, both classic and anaplastic EPNs display dot-/ring-like cytoplasmic positivity for EMA, strong positivity for GFAP (mostly in pseudorosettes), and sparse OLIG2 expression (Figure 1). The Ki67 proliferation index varies from low (classic EPN) to moderate/high (anaplastic EPN).

Myxopapillary EPNs are rare ependymal tumors, occurring almost exclusively in the lower spinal cord. Histologically, they consist of elongated, fibrillary processes radially arranged around vascularized, myxoid, or fibro-vascular cores. In rare cases, myxopapillary EPNs consist of confluent sheets of polygonal cells with little (if any) papillary growth pattern. Round eosinophilic, PAS-positive structures (referred to as “balloons”) are occasionally seen (Figure 1).

SEs are very indolent lesions that are typically intra-ventricular and are composed of clusters of small cells in a coarse or myxoid glial matrix. The proliferation index is typically low and mucoid degeneration is frequently documented. SEs are positive for GFAP, but (unlike classic EPNs) have patchy EMA staining (Figure 1) [4].

The grading of ependymal tumors is a matter of ongoing debate. Traditionally, SEs and myxopapillary EPNs are regarded as grade I tumors, classic EPNs as grade II, and anaplastic EPNs as grade III tumors [4]. However, an international panel of experts (cIMPACT working committee [WC] 2) very recently proposed relevant changes to this grading system. According to cIMPACT WC2, myxopapillary EPNs should be designated as grade II tumors due to their intrinsic potential for local and/or distant spreading. Moreover, the histological distinction between classic and anaplastic EPNs should be abandoned, given (i) its poor reproducibility, (ii) its limited prognostic impact, and (iii) the discovery of molecular signatures that surpass histological assessment [17]. This approach was made possible by the recent classification of ependymal tumors, based on each entity’s molecular features and anatomic distribution [18].

### 2.2. Molecular Classification of Ependymal Tumors

The genomic characterization of cancer has now become crucial for diagnosis, prognostic estimate, and treatment selection [19]. The classification system of primary brain tumors (PBTs) has historically been based solely on histopathologic features, with limited clinical utility due to the lack of reproducibility in predicting patients’ outcomes [20]. Several key genomic alterations have been identified over the last decades as a result of large-scale sequencing efforts, thrusting central nervous system (CNS) malignancies into a new “molecular era” [4,21]. These advances led to a major update to the WHO classification in 2016 wherein, in addition to histology, some of these molecular factors were introduced to define many PBT entities [4]. Methylation and gene expression studies have identified nine molecular groups of ependymal tumors across three CNS compartments (i.e., supratentorial [ST], posterior fossa [PF], and spinal cord [SC] region) (Table 1) [18,22,23,24,25,26].

Moreover, genes displaying hypermethylation in adults are involved in neurogenesis and embryo development; indeed, many HOX gene family associated with hindbrain development during early embryogenesis can be hypermethylated [27].

ST neoplasms include: (i) tumors with SE morphology (ST-SE), (ii) EPNs with recurrent C11orf95-RELA fusions (ST-EPN-RELA), and (iii) EPNs with YAP1-MAMLD1 fusions (ST-EPN-VAP7) [18] (Table 1). This molecular stratification has clinical and prognostic implications, since ST-EPN-RELA is associated with a worse prognosis than ST-EPN-VAP7 or ST-SE. In everyday clinical practice, RELA fusions may be assessed by means of FISH analysis (RELA break apart probes), or immunohistochemistry for p65/RELA and L1CAM [28].

PF neoplasms include: (i) tumors with SE morphology (PF-SE), (ii) group A EPNs (PF-EPN-A), and (iii) group B EPNs (PF-EPN-B) [18]. Unlike ST-EPNs, PF tumors lack recurrent gene fusions and are mainly distinguished by gene expression profiles. PF-EPN-A tumors are most common in infants and have a poor prognosis. PF-EPN-B are, instead, typical of older children/adults and have a more favorable outcome. The molecular stratification of PF-EPNs is performed either by means of DNA methylation studies or by H3K27me3 immunostaining. The latter is negative in most PF-EPN-A, representing a valid and cost-effective substitute for DNA methylation studies [29].

Finally, SC neoplasms include: (i) rare SE tumors (SC-SE), (ii) mixopapillary EPN (SC-MPE), and (iii) spinal EPNs (SC-EPN) [18]. These molecular categories recapitulate the histological subtypes of ependymal tumors. Rare cases of histologically defined classic EPNs, however, fall into SC-SE or SC-MPE molecular subgroups. This discrepancy’s clinical and biological significance is largely unknown [25,26,28,29,30]. Recent methylation studies have also highlighted cases of SC-EPNs with MYCN gene amplification that have an aggressive clinical course, diffuse leptomeningeal involvement, and survival curves comparable to those seen in ST-EPN-RELA and PF-EPN-A tumors. According to the cIMPACT WC2,these neoplasms should be considered as a novel molecular group (SC-EPN-MYCN) to be assessed using *MYCN* amplification assays in all newly diagnosed SC-EPNs [25,31,32]. In Table 1 are also reported the genetic characteristics, the more frequent gender and the outcome correlating to the specific subgroup of ependymoma.

### 2.3. Liquid Biopsy for the Detection of Ependymomas

The term “liquid biopsy” refers to non-invasive tools developed to detect and analyze tumor genetic material, such as circulating tumor cells (CTCs), cell-free circulating tumor DNA (ctDNA), extracellular vesicles, RNA, and noncoding miRNA obtained primarily from peripheral blood and from a variety of biofluids [33]. Given their potential to provide the entire genetic landscape of cancer lesions and to monitor clonal evolution over time, a liquid biopsy strategy has the potential to greatly aid each stage of PBT patient management, including early cancer detection, biomarker-driven therapies, minimal residual disease assessment, monitoring tumor burden, and response to oncological treatments [34]. Applying highly sensitive PCR-based techniques such as droplet digital PCR (ddPCR) and beads, emulsion, amplification, and magnetics (BEAMing) digital PCR, plasma ctDNA-based liquid biopsy has been already shown to be highly informative in many other solid tumors, tracking changes in tumor burden and mutational patterns. Apart from such targeted approaches, whole-genome or whole-exome sequencing (WGS, WES), which facilitate a comprehensive identification of genetic alterations without prior knowledge, have also been successfully implemented, despite their lower resolution and higher costs. The ideal source of circulating biomarkers in PBTs has been a subject of much debate. As for other solid tumors, peripheral blood was the first to be investigated, due to its natural advantages, such as quick and non-invasive collection. Two studies evaluating serum ctDNA in very large cohorts of patients with a wide range of tumor types, demonstrated that blood is not the optimal source for liquid biopsy in PBTs [35,36]. Bettegowda et al. used targeted sequencing, WES, or WGS to fully characterize plasma ctDNA from 640 patients with various cancer types and at different stages, including 41 cases of PBTs [35]. Although mutant DNA was found in the blood of most patients with advanced cancers (pancreatic, ovarian, colorectal, bladder, gastroesophageal, breast, melanoma, hepatocellular, and head and neck), less than 10% of the 27 patients with low and high-grade gliomas (LGGs; HGGs) and less than half of the 14 patients with medulloblastoma had detectable levels of ctDNA in their plasma [35]. Schwaderle et al. obtained the same results when they searched for ctDNA in 670 plasma samples of patients with different types of tumors, including 152 (22.7%) cases of PBTs, using digital next generation sequencing (NGS) with different-sized gene panels [36]. Only 32% of patients with PBTs showed at least one somatic mutation in the ctDNA. Among these mutations, 4% are associated with an FDA-approved drug and 11% with a novel agent under investigation, while 85% had non-actionable alterations [36].

Given its anatomical proximity to the brain parenchyma, and its usefulness for the diagnosis of other CNS pathological conditions, CSF has been investigated as an alternative source of ctDNA [37]. In 2015, Wang et al. studied the presence of ctDNA in the CSF of 35 patients with different PBTs and anatomical location (14 in the posterior fossa, 8 in the supratentorial compartment, and 13 in the spinal cord) [38]. The cohort of patients consisted of 10 LGGs, 13 HGGs, 6 medulloblastomas, and 6 ependymomas, including 3 spinal WHO grade II ependymomas, 2 spinal WHO grade I myxopapillary ependymomas, and 1 intracranial WHO grade II ependymoma [38]. Most CSF samples were collected directly from CNS cavities at the time of initial surgery [38]. Using targeted sequencing followed by WES, at least one mutation was identified in each of the 35 tumors [38]. Such mutations were found in 74% (95% CI: 57–88%) of the 35 matched CSF samples, showing a sensitivity comparable to that observed in bodily fluids adjacent to other tumor types, such as urine in urothelial cancer or bronchial washing in lung cancer [38,39,40]. The average detectable mutant allele fraction in CSF was 12.2%, lower than the fraction in tumor tissues but significantly exceeding the detection limit of the sequencing assay used (0.01%) [38]. In a multivariate logistic regression analysis, the proximity to CSF reservoirs and the tumor grade were the two clinical factors that strongly correlated with the detection of CSF ctDNA [38]. All tumors (13 WHO grade III or IV gliomas, 5 medulloblastomas, and 3 ependymomas; 100% of 21 cases) abutting a CSF reservoir or cortical surface had detectable levels of CSF ctDNA, whereas no ctDNA was present in the CSF of tumors encased in brain parenchyma (*p* < 0.0001) [38]. Interestingly, CSF ctDNA was found in 5 out of 6 cases of ependymomas, detecting mutations in genes ANKS3, HIST1H3C, TTC16, CDH5 and COL6A1 [38]. This study, along with other earlier experiences, suggested that CSF represents the most promising source of ctDNA, where a wide-range of genomic alterations, including putative actionable mutations and copy number alterations (CNAs) can be detected with high levels of sensitivity [34,38]. Subsequent studies were primarily focused on diffuse malignant gliomas and metastatic CNS lesions, either by using an NGS-based array for the comprehensive genomic characterization of CSF ctDNA, or by using a PCR-based targeted approach to search for known hotspot mutations with particular diagnostic or prognostic relevance [34,41]. One of the most significant contributions in this field was published by the Memorial Sloan-Kettering group in 2019 [41]. Miller et al. investigated whether a high-throughput sequencing assay (MSK-IMPACT) applied to ctDNA in CSF was able to characterize the glioma genetic landscape and track its evolution over time. 85 CSF samples from adult patients with diffuse gliomas of various grades (46 GBMs, 26 WHO grade III, and 13 WHO grade II) were collected through LP as part of patients’ clinical management for signs or symptoms indicative of CNS infection, leptomeningeal spread, or increased intracranial pressure [41]. Notably, the collection of CSF generally occurred well after initial surgery and, in all cases, after the completion of adjuvant oncological treatments [41]. A total of 42 out of 85 (49.4%) patients presented tumor-derived genetic alterations in their CSF, including telomerase reverse transcriptase promoter (TERTp), isocitrate dehydrogenase (IDH) mutations, homozygous deletions of cyclin-dependent kinase inhibitor A and B (CDKN2A/CDKN2B), epidermal growth factor receptor (EGFR) amplifications, and in-frame EGFR variant III deletion The detection of ctDNA in CSF was strongly associated with multiple radiological parameters, including tumor progression, tumor burden, and intraventricular spread, whereas no correlation was observed with grade, disease duration, or prior therapy [41]. This study’s findings, demonstrating that ctDNA may provide a comprehensive and genetically faithful representation of the corresponding tumor genome, are crucial because they may be extended to all PBTs besides malignant gliomas. Clinical experiences evaluating liquid biopsy strategies exclusively in patients with ependymomas are still lacking, with only small case series or case reports available in the literature [42,43,44]. In the brief case series by Connolly and collaborators, a small quantity of mutant signal for DDX41 was found in CSF ct-DNA of 1 out of 3 patients.

Despite the fact that available data on liquid biopsy in the field of CNS malignancies originate from a small and often heterogeneous series of patients, some important conclusions can be drawn. First, CSF seems to be the best source of genetic material for a liquid biopsy strategy, as blood tumor DNA levels are low and only detectable in a few patients, probably due to the presence of the BBB. Second, liquid biopsy may not be as informative across all PBT types. Leakage into genetic material’s CSF from tumors encapsulated in brain parenchyma, and not directly adjacent to a CSF reservoir or the cortical surface, appears to be very low and frequently undetectable. Moreover, given their slow growth-fraction rate and poor cellularity, only few low-grade tumors release detectable amounts of DNA into the CSF. Third, ctDNA may provide a comprehensive and genetically faithful representation of the corresponding tumor genome. Given that the vast majority of ependymomas develop within, or communicate directly with, a ventricular reservoir, a CSF ctDNA assay may be particularly appropriate for such tumors, making them ideal candidates for CSF monitoring.

## 3. The Role of Surgical Treatment

According to major studies [45], surgery is the first and most critical treatment for intracranial ependymomas, since the extent of resection is one of the most significant predictors of outcome (see Table 2). Based on the principle of “onco-functional balance”, the goal is to achieve a maximally safe resection without neurological impairment. It is, therefore, necessary to delineate and monitor the motor or sensory regions to preserve their structural and functional integrity. Intraoperative neurophysiological monitoring is employed for this purpose. Neuronavigation with tractography is another intraoperative technique used to maximize the extent of resection while maintaining the patient’s neurological integrity, given that ependymomas tend to displace white matter tracts. 5-ALA-induced-fluorescence may be used to improve the extent of surgical resection, making it possible to clearly differentiate normal tissue from the tumor in the area of origin, despite the fact that the use of 5-ALA is actually off-label when used for tumors other than malignant gliomas. A recent review [46] described the treatment of 7 ependymomas with 5-ALA (5 intracranial of the IV ventricle and 2 intramedullary), demonstrating that tumors were fluorescent in all cases. Hence, 5-ALA might be very helpful, given the priority of distinguishing between highly eloquent healthy parenchyma and a tumor. Grade I ependymal tumors tend to be well-demarcated, and complete surgical excision is typically curative [2], whereas grade II and III intracranial tumors are commonly treated by means of maximal surgical excision [14] followed by oncological treatment.

### 3.1. Supratentorial Ependymomas

Supratentorial tumors (ST) account for 19.3–34% [12,47] of ependymomas and are usually in contact with a ventricular surface growing into the brain parenchyma. However, as stated above, they may arise from ependymal rests within the parenchyma and tend to be relatively well-demarcated from the surrounding brain. The transcortical approach may be used, given that the majority of supratentorial extraventricular ependymomas are located in the frontal or parietal lobe [48], whereas an interhemispheric transcallosal approach is more commonly preferred in the case of intraventricular lesions. Intraoperative neurophysiology monitoring is advisable. Gross total resection for ST ependymoma is feasible and has been reported in a high percentage of cases (about 75%) [13,49]. Nevertheless, supratentorial ependymomas generally have a higher tumor grade than infratentorial ependymomas, which results in poorer progression-free survival and overall survival [48]. A retrospective series of 46 ST ependymomas [49] was conducted to characterize the roles of surgery and histology in tumor control, demonstrating that age, the extent of resection, and histologic grade are the most significant prognostic factors affecting the outcome of patients with ST tumors. Adjuvant RT was administered to patients with grade III ependymoma, following gross total or subtotal resection. The 5- and 10-year overall survival rates for the entire population were 57.1% ± 8.7% and 41.8% ± 9.9%, respectively. The 5- and 10-year progression-free survival rates for the entire cohort were 33.8% ± 8.1% and 25.4% ± 8%, respectively.

### 3.2. Infratentorial Ependymomas

The majority of intracranial (IT) ependymomas are located in the infratentorial region (36–80.7%) [5,47], in the posterior fossa and principally in the fourth ventricles. Fourth ventricle tumors most commonly originate from the caudal floor and project up into the ventricle. Infratentorial ependymomas have a worse prognosis due to their propensity to invade the obex, which may preclude complete removal. Reported data for infratentorial tumors in adults with an intracranial ependymoma suggest that gross total resection (GTR) is feasible in a lower percentage (42%) [48] than it is for supratentorial ependymoma, since gross total resection may not be possible when the tumor invades the floor of the IV ventricle extensively or extends through the foramen of Luschka (risk of bradycardia) [1]. Furthermore, the encasement of cranial nerves and brainstem vasculature may limit resectability [45]. Persistent hydrocephalus despite tumor resection requires shunting or endoscopic ventriculostomy

**Figure 2:**
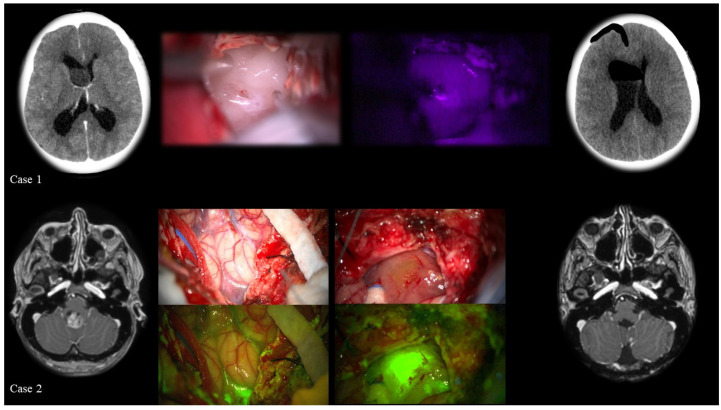
Supratentorial (case 1) and infratentorial (case 2) ependymomas. Case 1: a 50-year-old female patient with a lesion in the frontal horn of the right lateral ventricle: a right fronto-parietal transcortical approach was used to remove the tumor whose histology, based on the WHO classification, was A grade I ependymoma). From left to right: pre-operative CT scan; intraoperative image of the tumor under white light microscope illumination; blue light illumination using 5-ALA: in this case the tumor was not fluorescent; and finally, the post-operative CT scan. Case 2: a 29-year-old female patient with an intraventricular lesion in the IV ventricle, with moderate contrast enhancement in T1-weighted MRI scan. The histological diagnosis revealed a grade III ependymoma (WHO 2016).

In a retrospective series of WHO grade II ependymomas in adults, the 5- and 10-year overall survival (OS) rates were 86.1% and 81%, respectively, when 80.7% of infratentorial and 19.3% of supratentorial ependymomas were considered [47]. Preoperative Karnofsky Performance Status (KPS), the extent of resection, and tumor location were independent prognostic factors for OS. We reported two cases of ependymoma patients undergoing surgery in Figure 2.

**Table 2:** Important studies evaluating the role of surgery in ependymoma patients. IT: infratentorial; ST: supratentorial. GTR: gross total resection; STR: subtotal resection; y = years; NA = not available.

Study	N.	Location	Grading	EOR	OS	PFS
Varma, 2018 [50]	13	IT 61.5% ST 38.5%	I	GTR 92% STR 8%	NA	NA
Song, 2017 [15]	53	IT 64.2% ST 35.8%	II 66% III 34%	GTR 54.7% STR 45.3%	5 y 82.5%, 10 y 75.7%	NA
Dutzmann 2013 [5]	64	IT 35.6% ST 34.4%,	I 28.1% II 51.6% III 20.3%	GTR 76.6%	NA	NA
Metellus, 2010 [47]	114	IT 80.7% ST 19.3%	II	GTR 58.7%, STR 41.3%	5 y 86% 10 y 81%	5 y 74.6% 10 y 58.9%
Vitanovics, 2009 [51]	61	IT 51% ST 49%	II 65.5% III 34.5%	GTR 60% STR 40%	NA	NA
Figarella-Branger, 2007 [13]	216	IT 66% ST 34%	II 73% III 27%	NA	NA	NA
Metellus, 2007 [12]	152	IT 70% ST 30%	II 72% III 28.3%	GTR 58.6% STR 41.4%,	5 y 84.8% 10 y 76.5%	5 y 63.5% 10 y 52.8%
Metellus, 2007 [49]	121	IT 66% ST 34%	II 72.7% III 27.3%	GTR 63% STR 37%	5 y 85% 10 y 76%	NA
Reni, 2003 [52]	70	IT 44% ST 56%	II 77% III 23%	GTR grade II 63% GTR grade III 47%	5 y 67% 10 y 50%	NA
Donahue, 1998 [53]	10	IT 80% ST 20%	not specified	GTR 10% STR 90%	NA	NA

## 4. The Role of Radiation Therapy

Consensus exists regarding the inclusion of postoperative radiotherapy (RT) in the standard of care for adult patients with an anaplastic ependymoma classified as grade III and grade II (after an incomplete resection) by the World Health Organization (WHO) [45,54]. On the other hand, the role of postoperative RT in patients with grade II ependymoma who undergo complete resection remains controversial [55].

Due to a lack of level I evidence, retrospective studies were used to justify the recommendation of adjuvant RT for adult patients with an ependymoma. However, the results of RT are ambiguous in terms of overall survival (OS), due to the retrospective nature of the studies, the small sample size, and disparate results (see Table 3).

Two large retrospective studies involving patients with intracranial WHO grade II ependymomas showed no significant benefits in terms of progression-free survival (PFS) and OS when RT was scheduled for the entire study population [16,47]. However, Metellus et al. reported that postoperative RT improved PFS and OS in the subgroup of patients with incomplete resection [47]. While the evaluation published by Nuño et al. using the National Cancer Database (NCDB) demonstrated no advantage in the use of RT for grade II–III adult ependymomas, regardless of tumor grade or extent of resection [16], there were several biases: the clinical characteristics between patients receiving RT or observation were not evaluated, and no data were reported concerning the dose and fractionation of RT.

Other investigations, based on the SEER program and NCDB, showed that postoperative RT is only beneficial for children with grade II-III ependymoma, including those with grade II ependymomas with subtotal resection, and not for adults [56,57]. However, the SEER and NCDB findings should be interpreted with caution due to missing data, which prevented an accurate evaluation of therapeutic approaches.

In a univariate analysis of 152 adult patients, the French Society of Neurosurgery and Neuro-Oncology also reported a significant improvement in survival with RT: the 5-year OS rate increased from 73.6% to 93.1% [12].

In a multi-institutional retrospective study of 172 adults with WHO grade II–III ependymomas, Woo Wee et al. observed a significant improvement with postoperative RT [58]. A multivariate analysis of postoperative RT showed a marginal OS benefit across all the population study, although a specific subgroup that may benefit the most from this treatment was not defined.

Furthermore, in the subgroup of 106 patients with grade II, multivariate analysis revealed that RT significantly improved local control and PFS. Although no obvious benefit in terms of OS was observed in these patients, the 5- to 10-year OS rate was 89.6–87.8%.

Other studies concerning the subgroup of patients with Grade II reported similar results, with an increase in PSF and OS following RT [52,59].

Only one prospective observational study for adult ependymomas, undertaken by the Collaborative Ependymoma Research Network (CERN Foundation), has been published to date [60], with no PFS benefit. However, the authors reported superior PFS in patients with infratentorial grade II–III ependymoma treated with GTR and RT versus GTR alone. In multivariate analysis, the worst PFS was reported in patients with subtotal resection without adjuvant RT [60].

As regards target volume irradiation, while earlier studies suggested that patients might benefit from craniospinal RT, it has recently been indicated that irradiating the local field achieves good local control with low risk of spinal dissemination [45]. Based on the latter background, stereotactic radiosurgery (SRS) was used for incompletely resected recurrent ependymomas or initially unresectable ependymomas [61]. SRS is capable of delivering a single higher ablative dose of radiation to the target volume with a rapid radiation fall-off. Despite the paucity of available data on SRS, the above-mentioned published analysis reported a 1-3-5 year OS of 60%, 36%, and 32%, and a 1-3-5 year PFS of 82%, 46%, and 46%, respectively [61].

Another interesting RT scenario is proton therapy (PT), which provides dosimetric advantages due to steep dose fall-off, decreased integral dose to normal brain, and a lower risk of side effects. The recent analysis of Stross et al. showed a substantial increase in the use of PT for medulloblastoma and ependymoma in the pediatric population [62]. Clearly, the data can be extrapolated to the adult population, but no firm results in this field have been published.

To conclude, adjuvant RT appears to be beneficial for grade II-III ependymoma in terms of disease control and survival. However, the subgroup of patients who would benefit the most needs to be further identified. Nevertheless, the contradictory results highlight the necessity for high-quality prospective studies to guide treatment recommendations in adult ependymoma.

## 5. The Role of Systemic Treatments

In children younger than 18 months, initial treatment with chemotherapy alone is an option to defer or avoid radiotherapy [45]. In adults, however, chemotherapy is currently reserved for patients with recurrent disease who are no longer candidates for re-surgery and re-irradiation [45]. Except for one recent phase II study, studies addressing the role of chemotherapy in adults with recurrent ependymomas have been limited to small retrospective series or case reports (see Table 4).

## 6. Chemotherapy for Recurrent Intracranial Ependymomas

In the early 2000s, two retrospective studies found that platinum-based regimens were associated with a higher response rate compared to nitroso-urea based regimens in adults with recurrent intracranial ependymomas [71,72]. However, there was no difference in terms of PFS. In these studies, the response rate ranged from 21% to 30%, and the median PFS was between 6 to 10 months [71,72]. Several studies subsequently explored the role of temozolomide (TMZ) [64,66,67,70]. In a retrospective study of 18 patients, TMZ alone (standard schedule) achieved a response rate of 22% and a median PFS of 9.7 months. Responses were not associated with the *MGMT* methylation status and were only observed in chemotherapy-naïve patients [65]. To corroborate this last finding, TMZ alone had no efficacy in a retrospective study of 25 patients with recurrent grade II ependymomas who progressed following first-line platinum-based chemotherapy (4% response rate, median PFS of 2 months) [69]. A recent phase II study, conducted within the framework of the Collaborative Ependymoma Research Network, evaluated dose-dense TMZ in combination with lapatinib [63]. Fifty adult patients with recurrent intracranial and spinal grade I, II, and III ependymomas were included in the study. The rationale for the dose-dense TMZ schedule was to target the unmethylated *MGMT* promoter in ependymomas, since dose-dense TMZ may decrease the level of MGMT. Lapatinib, an epidermal growth factor receptor inhibitor, was used because of its ability to inhibit both ErbB2 (human epidermal growth factor receptor 2) and ErbB1 (epidermal growth factor receptor), which are frequently overexpressed in ependymomas. The combination of dose-dense TMZ and lapatinib resulted in a 16% response rate and a median PFS of 7.8 months. Efficacy was not associated with tumor location or grade. Treatment was well tolerated and it is interesting to note that most patients reported a clinical benefit. The efficacy of bevacizumab (alone or in combination) was addressed in a small retrospective study [68]. Although the response rate was 75%, median PFS was similar to that reported with platinum-based or TMZ-based regimens. Small studies on recurrent adult spinal ependymomas provide some information concerning systemic treatments. In a series of 10 patients, etoposide resulted in a 30% response rate and a median PFS of 15 months [77]. The median PFS in patients with recurrent spinal ependymomas treated with a dose-dense TMZ and lapatinib combination was 7.5 months [63]. Bevacizumab has shown some efficacy in spinal cord ependymomas occurring in NF2 patients, especially when these tumors harbor an important cystic component [78], and can also result in some response in recurrent spinal cord ependymomas [75]. Moreover, prolonged response to a checkpoint inhibitor was recently reported in a patient with a metastatic myxopapillary ependymoma [76].

## 7. Future Perspective

Overall, recurrent ependymomas in adults appear to be moderately chemosensitive tumors. In children, studies in the recurrent setting have also reported low response rates with standard chemotherapy, high-dose chemotherapy, bevacizumab-based regimens, and targeted therapies such as erlotinib and sunitinib [45]. Future studies will be required to determine whether response to chemotherapy and personalized treatments are associated with recently identified molecular subgroups of ependymomas. Whether recurrent ependymomas may benefit from immunotherapy is also a matter of debate.

## Figures and Tables

**Table 1:** Clinical and molecular characteristics of ependymomas based on tumor location.

Anatomical Location	Molecular Subgroup	Genetic Characteristics	Histopathology (WHO Grade)	Age	Gender	Outcome
Supratentorial (ST)	SE	Balanced	Sub-ependymoma (WHO I)	Adulthood	>M	Good
	EPN-YAP1	Aberr. 11q	Classic/Anaplastic (WHO II-III)	Infancy to childhood	>F	Good
	EPN-RELA	Aberr. 11q	Classic/Anaplastic (WHO II-III)	Infancy to childhood	>M	Poor
Posterior fossa (PF)	SE	Balanced	Sub-ependymoma (WHO I)	Adulthood	>M	Good
	EPN-A	Balanced	Classic/Anaplastic (WHO II-III)	Infancy	>M	Poor
	EPN-B	CIN	Classic/Anaplastic (WHO II-III)	Childhood to Adulthood	>F	Good
Spinal (SP)	SE	6q del.	Sub-ependymoma (WHO I)	Childhood to Adulthood	M = F	Good
	MPE	CIN	Mixopapillary Ependymoma (WHO I)	Adulthood	M = F	Good
	EPN	CIN	Classic/Anaplastic (WHO II-III)	Adulthood	>M	Good

SE = subependymoma; EPN = ependymoma; MPE = mixopapilalry ependymoma; M = males; F = females.

**Table 3:** Most relevant studies investigating the role of RT in ependymal tumors. RT = radiation therapy; PFS = progression-free survival; OS = overall survival; NA = not available; pts = patients.

Study	Trial Design	N° of pts	Median Age (Range)	Grading and Tumor Site	N. of pts Treated with RT	Efficacy
Metellus P et al., 2010 [47]	Retrospective	114	48 (18–82)	WHO grade II intracranial ependymoma	35	5-year OS: Surgery: 83.4% Surgery plus RT: 92%
Nuño M et al., 2016 [16]	Retrospective; USA National Cancer Database	1055 Grade II 263 Grade III	44 (31–56)	WHO grade II/III supratentorial and posterior fossa ependymoma	662	RT does not seem to have an impact on overall survival
Deng X et al., 2020 [56]	Retrospective; SEER database	560 Grade II 163 Grade III	Range (18–68)	Intracranial WHO grade II/III ependymoma	422	RT does not seem to have an impact on overall survival
Prabhu RS, et al., 2020 [57]	Retrospective; SEER database	1787	45–50 (37–62)	WHO grade II/III ependymoma	856	3- and 5-year OS with adjuvant RT was 83.4% and 79.3% versus 86.4% and 81.8% with observation.
Woo Wee et al., 2020 [58]	Retrospective; Multicenter retrospective	172	NA	WHO grade II/III ependymoma	110	5- and 10-year OS rates were 76.6%/71.0%, respectively. PORT significantly elevated the rates of PFS (*p* = 0.002), and OS (*p* = 0.043)

**Table 4:** Summary of studies and case reports analyzing the impact of chemotherapy in recurrent adult intracranial and spinal ependymomas. PFS = progression-free survival; OS = overall survival; ST = supratentorial; IF = infratentorial; SP = spinal; CR = complete response; PR = partial response; MR = minimal response; RT = radiation therapy; TMZ = temozolomide; PCV = procarbazine, carmustine, vincristine; MPE = myxo-papillary ependymoma.

	N	Year	Study Type	Age	Chemotherapy	Grade (%)	Tumor Location (%)	Response Rate (%)	Median PFS (Months)	Median OS (Months)
Recurrent intracranial ependymomas										
Gilbert [63]	50	2021	Phase II	43.5	Dose-dense TMZ + lapatinib	I (16%) II (32%) III (40%)	ST (30%) IT (16%) SP (51%)	CR (4%) PR (12%)	7.8	27
Gramatzki [64]	17	2016	Retrospective	28	TMZ, PCV, platinum-based, epirubicine plus ifosfamide	II (23%) III (77%)	ST (65%) IT (35%)	CR (6%) PR (6%)	6	41
Ruda [65]	18	2016	Retrospective	42	TMZ	II (45%) III (55%)	ST (61%) IT (39%)	CR (5%) PR (17%)	9.7	30.5
Lombardi [66]	1	2013	Case report	45	Cisplatin + TMZ	III	ST	PR	9	11
Freyschlag [67]	1	2011	Case report	25	TMZ	III	ST	PR	5+	5+
Green [68]	8	2009	Retrospective	40	Bevacizumab alone or in combination	II (38%) III (62%)	ST (75%) IT (25%)	PR (75%)	6.4	9.4
Chamberlain [69]	25	2009	Retrospective	49	TMZ in platinum-refractory tumors	II (100%)	ST (100%)	CR (0%) PR(4%)	2	3
Rehman [70]	1	2006	Case report	24	TMZ	I	IT	CR	120	120
Brandes [71]	28	2005	Retrospective	44	Cisplatin-based (46%) Non cisplatin-based (54%)	II (61%) III (39%)	ST (54%) IT (46%)	CR (7%) PR (14%)	9.9	40.7
Gornet [72]	14	1999	Retrospective	31	Platinum-based Nitroso-urea based	II (50%) III (37%)	ST (37%) IT (37%) SP (24%)	PR (12%) MR (31%)	3–10	-
Recurrent spinal cord ependymomas										
Gilbert [63]	50	2021	Prospective	43.5	Dose-dense TMZ + lapatinib	I (16%) II (32%) III (40%)	ST (30%) IT (16%) SP (51%)	CR (4%) PR (12%)	7.8	27
Tapia Rico [73]	1	2020	Case report	25	Tislelizumab (anti-PD1)	Metastatic MPE	SP	Stable disease	18	28+
Fujiwara [74]	1	2018	Case report	26	TMZ	Metastatic MPE	Spinal	CR	72+	72+
Lorgis [75]	2	2012	Retrospective	45	Cisplatin, cyclophosphamide, bevacizumab	III (100%)	SP (100%)	PR (100%)	12+	12+
Kim [76]	2	2011	Case report	26	RT + TMZ	III (50%)	SP (100%)	-	3–36+	12–39+
Chamberlain [77]	10	2002	Prospective pilot study	30	Etoposide	Low-grade (100%)	SP (100%)	PR (20%)	15	17.5
